# Repeat Testing Reliability of Plasma pTau217 in Preclinical AD

**DOI:** 10.1002/alz.092759

**Published:** 2025-01-09

**Authors:** Robert A. Rissman, Oliver Langford, Rema Raman, Michael C. Donohue, Doris P. Molina‐Henry, Sara Abdel‐Latif, John R. Sims, Roy Yaari, Karen Chilcott Holdridge, Keith A Johnson, Paul S. S. Aisen, Reisa A Sperling

**Affiliations:** ^1^ Alzheimer's Therapeutic Research Institute, University of Southern California, San Diego, CA USA; ^2^ Eli Lilly and Company, Indianapolis, IN USA; ^3^ Gordon Center for Medical Imaging, Massachusetts General Hospital, Harvard Medical School, Boston, MA USA; ^4^ Center for Alzheimer’s Research and Treatment, Brigham and Women’s Hospital, Massachusetts General Hospital, Harvard Medical School, Boston, MA USA

## Abstract

**Background:**

Data from the Anti‐Amyloid Treatment in Asymptomatic Alzheimer's Disease (A4) trial (NCT02008357), including cognitively unimpaired participants with brain amyloid pathology on florbetapir F 18, demonstrates that levels of plasma P‐tau217, as detected by an electrochemiluminescence (ECL) immunoassay, is strong predictor of elevated cerebral amyloid on florbetapir PET in cognitively unimpaired individuals. Here we compare plasma P‐tau217 measures over 12 weeks using a P‐tau217 ECL immunoassay.

**Method:**

A4 trial placebo‐group participants who had their first baseline and first post‐baseline plasma P‐tau217 samples collected within 175 days of each other were included in these analyses. This time was chosen to maximize the number of available samples while minimizing the likelihood of significant change. The coefficient of variation (CV) was used to measure relative variability between samples computed as the difference in plasma P‐tau217 values, and the “test‐retest” performance of the plasma P‐tau217 assay was evaluated using an intraclass correlation coefficient (ICC) from a two‐way mixed‐effects model. A Box‐Cox data transform was performed due to significant observed heteroscedasticity in the P‐tau217 values.

**Result:**

Analysis sample included 573 A4 study participants (mean age: 71.9 +/‐ 5 years, mean florbetapir SUVr: 1.33 +/‐ 0.18; days between assays: 86.5 +/‐ 6.5 days; 59.9% female sex). Low levels of variability were observed between the samples with a coefficient of variation of 7% and the test‐retest reliability was ICC=0.90 (ICC: 0.90; 95% CI: 0.89, 0.92).

**Conclusion:**

A P‐tau217 ECL immunoassay showed low levels of variability between samples and excellent test‐retest reliability over 12 weeks in cognitively unimpaired individuals with elevated cerebral amyloid.

